# Optical detection of individual ultra-short carbon nanotubes enables their length characterization down to 10 nm

**DOI:** 10.1038/srep17093

**Published:** 2015-11-25

**Authors:** Zhenghong Gao, Laura Oudjedi, Romain Faes, Fabien Moroté, Christèle Jaillet, Philippe Poulin, Brahim Lounis, Laurent Cognet

**Affiliations:** 1 Univ. Bordeaux, LP2N, F-33405 Talence, France; 2 Institut d’Optique & CNRS, LP2N, F-33405 Talence, France; 3 CRPP, CNRS, Pessac, France; 4 LOMA, CNRS & Univ. Bordeaux, F-33405 Talence, France

## Abstract

Ultrashort single-walled carbon nanotubes, i.e. with length below ~30 nm, display length-dependent physical, chemical and biological properties that are attractive for the development of novel nanodevices and nanomaterials. Whether fundamental or applicative, such developments require that ultrashort nanotube lengths can be routinely and reliably characterized with high statistical data for high-quality sample production. However, no methods currently fulfill these requirements. Here, we demonstrate that photothermal microscopy achieves fast and reliable optical single nanotube analysis down to ~10 nm lengths. Compared to atomic force microscopy, this method provides ultrashort nanotubes length distribution with high statistics, and neither requires specific sample preparation nor tip-dependent image analysis.

Ultrashort single-walled carbon nanotubes (usCNTs) hold unique physical, chemical and biological properties that can be used for applications in diverse areas. In condensed matter physics, both theory and experiments have shown that the nanotube electronic band-gap increases as their length shortens down to tens of nanometers in response to quantum confinement effect along the length axis^1^. In nano-electronics, sub-10 nm carbon nanotube transistors showed optimal low voltage operation by comparison to any similarly scaled device suggesting that further optimization of carbon nanotube transistors could be valuable for future applications^2^. In materials engineering, usCNTs represent promising strategies for fabricating high-performance polymer-nanotube nanocomposites like biological tissue scaffolds^3^. They can be easily processed as low viscosity solutions in contrast to suspensions of long nanotubes^4^. Shortening nanotubes is also an efficient approach for improving gas or ion storage capabilities of CNT based materials^5^. In nanomedicine, usCNTs have been applied as gene delivery agents into cells where the gene expression could be modulated by the lengths of the nanotubes^6^. More recently, usCNTs were also shown to insert in the lipid bilayers of cell membranes behaving as biomimetic nanopores^7^ or artificial channels selectively transporting signaling and functional molecules and/or ions across cell membranes^8^. Their potential for biomedical applications is also strengthened by their easy extraction through the kidneys and bile ducts^9^.

For all these applications, it is becoming necessary to routinely characterize the lengths of usCNTs with precision and statistics, which is not yet achievable with current analytical methods. This would allow developing rational protocols for preparing usCNT samples and optimizing their length-dependent properties.

By removing the averaging inherent to ensemble measurements, the characterization of sample contents at the single (nano-)object level has proven to be key. It indeed provides a measure of the full distributions of object properties, and quantitatively reveals heterogeneities. Electron microscopy and atomic force microscopy (AFM) are the most commonly employed techniques for morphological characterization of nanostructures including nanotubes. For the smallest nano-objects, electron microscopy and AFM however require delicate sample preparation and extensive operation time^10^. These methods are also restricted to the analysis of very small sample fractions and therefore provide low throughput. In principle, optical microscopy can surpass these techniques in terms of throughput and ease of sample preparation, but this requires that single usCNTs can be reliably detected with an optical method.

Several optical microscopy techniques are commonly used to detect single-wall carbon nanotubes (SWCNTs). Raman scattering microscopy is the most widely applied optical method for carbon nanotube characterization both for semiconducting and metallic species^11^,^12^,^13^. The correlation between the intensity ratio of the disorder-induced D to G Raman bands and the inverse of the tube length has been proposed for the estimation of the average nanotube lengths in bulk samples down to 50 nm^14^. Raman scattering is however constrained by the weakness of the signals and the need for near-resonant tunable lasers such that detection at the single tube level of small diameter species is challenging, especially if one aims at studying usCNTs. Luminescence measurements, which are limited to semiconducting species, are also valuable to characterize SWCNT samples^15^. The luminescence yield is however extremely sensitive to nanotube backbone integrities. In particular, the shortening of nanotube lengths creates a number of defect sites (including at the nanotube ends) such that SWCNT luminescence vanishes when the distance between defect sites becomes shorter than the exciton diffusion lengths^16^,^17^. This implies that it is challenging to detect luminescent SWCNTs shorter than typically 100 nm at the single tube level. Because light absorption properties of carbon nanotubes are much less sensitive to the presence of defects, we postulate here that single nanotube measurements based on absorption should provide the best strategy for characterizing usCNT samples. Transmission spatial modulation techniques allowed the direct measurement of the extinction of long small-diameter SWCNTs, which is dominated by absorption for small-diameter nanotubes. Using high frequency modulation, an absorption cross-section of 3.2 × 10^−17^ cm^2^/C atom was precisely determined for (6,5) nanotubes at their second-order optical transition (S_22_)^18^. Such direct absorption measurements performed on single SWCNTs are however arduous because the weak signals have to be extracted from laser intensity fluctuations and contributions from environment scattering. Here we consider the use of photothermal imaging (PhI)^19^,^20^ to detect usCNTs. PhI is an indirect absorption detection modality, which achieves high detection sensitivity of absorbing nano-objects and is insensitive to scattering environments. It is based on the detection of refractive index variations that are induced by photothermal effect in the local environment of an absorbing nano-object^19^,^20^. PhI microscopy showed the possibility to image several types of absorbing nano-objects, including metallic or semi-conducting SWCNTs^21^ and reached unprecedented sensitivities for detecting tiny absorbers with absorption cross-sections as small as^19^,^22^,^23^ a few Å^2^. Considering the case of a small diameter 10 nm long (6, 5) SWCNT excited at its S_22_ resonance, a simple calculation would indicate an absorption cross-section of the order of a few 10^−14^ cm^2^. This simple calculation however raises the question whether PhI might provide a quantitative measure of usCNT lengths in different sample preparations.

In this work, we demonstrate that indeed, PhI allows the robust detection of usCNTs down to the range of 10 nm length and we show that the measured PhI signal amplitudes are proportional to nanotube lengths. In addition, by comparison with AFM, we demonstrate that PhI microscopy reliably provides fast characterization of usCNT samples with higher statistics.

## Results

**Figure 1:**
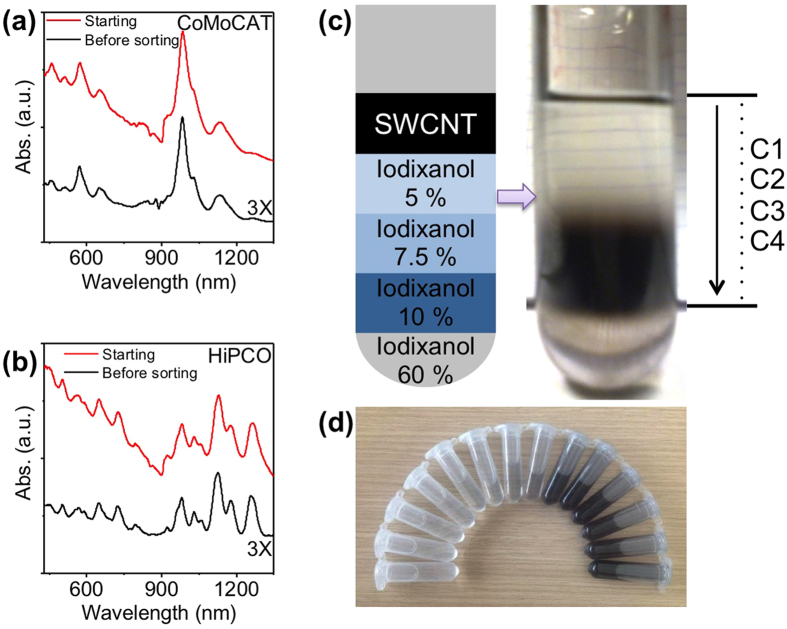
Absorption spectra of SWCNTs for CoMoCAT (a) and HiPco (b) samples. Red: starting SWCNTs in an aqueous solution of DOC; black line: usCNT suspension before DGU sorting; (**c**) Sample in centrifuge tube before (schematics) and after (photograph) DGU sorting. (**d**) length-separated CoMoCAT fractions collected after DGU.

Several methods have been proposed to produce usCNTs from raw nanotube materials. For instance, nanotube cutting and oxidization can be performed simultaneously using a mixture of sulfuric and nitric acids^4^,^24^ or using Piranha solution^25^. This approach is interesting for mass production of usCNTs, with rather broad length distribution, but it introduces various functional groups (e.g., -COOH, -OH, -C=O, and a small content of sulfur and nitrogen-containing groups) at the open ends and defect sites^4^. These degrade the sidewall structure integrity of the cut SWNTs and might affect their intrinsic properties. In order to obtain non-functionalized usCNTs, Dai *et al.* proposed an approach based on heavy sonication of fluorescein-polyethylene glycol coated SWCNTs^26^. The length distribution was further narrowed using density gradient ultracentrifugation (DGU)^27^. We prepared length-separated usCNT samples by slightly simplifying this protocol, to avoid the specific need for fluorescein-polyethylene glycol surfactant. In brief, we mechanically cut raw SWCNTs (HiPco or CoMoCat) into short ones by applying a tip-type ultrasonication in nanotube suspension containing 0.3 wt% sodium deoxycholate (DOC) as a dispersant (Fig. 1a,b) (see methods). DOC was chosen for its strong ability to interact with carbon nanotubes leading to high concentration carbon nanotube dispersions in water and bearing optimal optical properties^28^,^29^. Length separation of the cut nanotube samples was performed by DGU: a volume of 5 mL cut SWCNT solution was placed on the top of a density-gradient iodixanol solution and centrifuged at a speed of 45K rpm for 3 h (see methods). Upon centrifugation, SWCNTs with different lengths are spread at different heights along the centrifugal tube^27^ (Fig. 1c). The suspension containing length-separated usCNTs was then carefully divided and collected as fractioned samples along the tube from top to bottom (Fig. 1d). Both HiPco and CoMoCAT usCNT samples were prepared using this protocol. They were called herein C1 to C5 for CoMoCAT and H1 to H4 for HiPco nanotubes with increasing number corresponding to increasing length populations.

We then evaluated the content of usCNT fractions with PhI microscopy. A small amount of usCNTs suspensions was spin-coated onto clean microscope cover slips (see methods). Dilution factors and spinning rate were chosen to give a final usCNT surface density of less than 1 μm^−2^. A drop of viscous silicone oil was added on top of the samples to improve heat diffusion homogeneity at the glass-nanotube interface. The setup assembled for this study consists of a probe beam (HeNe laser, 633 nm, power of ~8 mW at the sample) overlaid with a cw absorption beam which intensity is modulated (Fig. S1). For the latter we used two different lasers with wavelengths either resonant to the S_22_ absorption band of (6,5) nanotubes (568 nm) or away from any optical resonance for the majority of the nanotubes present in the HiPco and CoMoCat samples (532 nm). The absorption and probe beams were both focused onto the sample using a high NA objective (60x, NA = 1.49). The absorption beam had an intensity of ∼*500* *kw/cm*^2^ at the sample and was circularly polarized to ensure that nanotubes are equally excited regardless of their orientation in the sample plane. Heat elevation induces time-modulated variations of the refractive index around an absorbing usCNT. The interaction of the probe beam with this index profile produces a scattered field with sidebands at the modulation frequency. The scattered field is then detected through its beatnote with the probe field reflected at the glass sample interface, which plays the role of a local oscillator as in any heterodyne technique and is extracted by lock-in detection. The modulation frequency was set to 700 kHz for which high signal to noise ratios are obtained with ms integration times^20^. The sample was mounted on a piezo-scanner stage that allowed scanning the sample to acquire 2D images. All data where acquired with integration times of 5 ms/pixel using a resolution of 100 nm/pixel.

**Figure 2:**
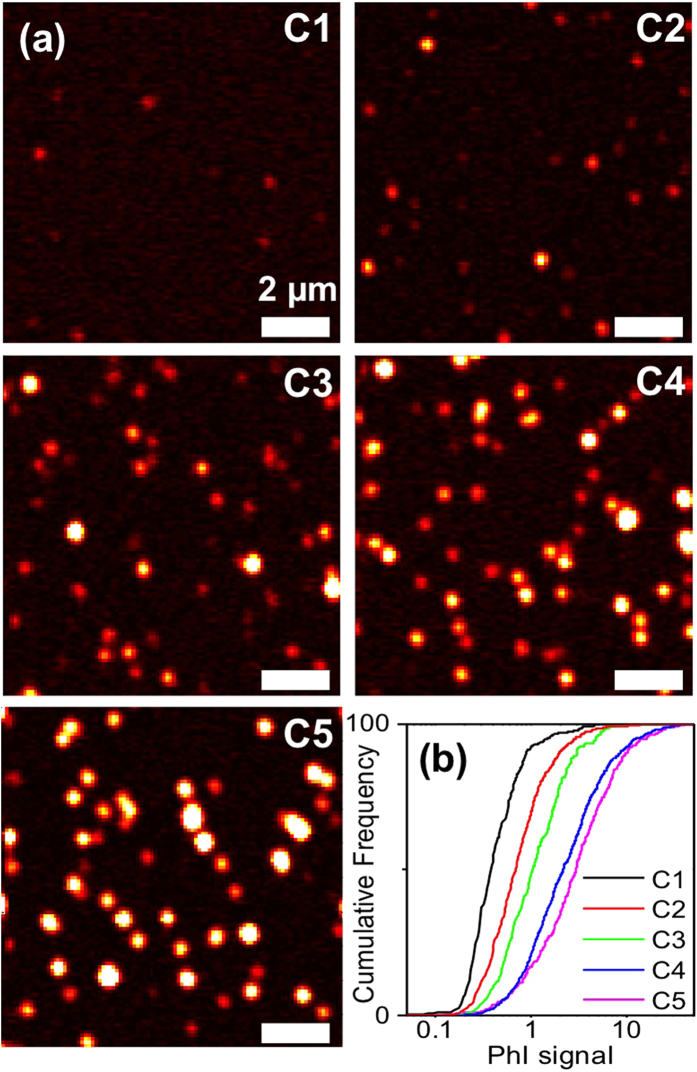
(**a**) PhI images of usCNTs in fractions C1, C2, C3, C4, and C5 (excitation at 568 nm). (**b**) Cumulative distributions of PhI signals arising from single usCNTs. All images were displayed with the same intensity scale.

Figure 2a shows examples of PhI images corresponding to fractions C1, C2, C3, C4 & C5 (CoMoCAT sample preparations). Typically 10 to 100 usCNTs can be detected per 10 μm × 10 μm image, corresponding to a detection rate of the order of one tube per second. For such images, 568 nm excitation was used to preferentially excite the most abundant (6,5) carbon nanotubes in CoMoCat samples^30^ (Fig. 1a). For fractions C1-C4, all signals appear as isolated spots, indicating that individual usCNTs are detected. The point-spread function of our microscope, primarily given by the product of the two focused beam spatial distributions^20^, is of the order of 230 nm. The observation of point-like and isolated signals indicates that in these short fractions, all nanotubes have lengths significantly shorter than 200 nm. Clearly, different signal intensities are found in the different samples, with signal amplitude increasing with fraction lengths. We analyzed the PhI signals by an automated home-built matlab routine to identify each point-like signal and fit its distribution by a 2D Gaussian. The fit amplitude of each spot provides a measure of the absorption signal of each detected nanotube.

In order to determine whether PhI signals are proportional to the usCNT lengths, we first plot in Fig. 2b the cumulative distribution of PhI signals for fractions C1, C2, C3, C4 & C5 (N = 300, 833, 397, 1188, 712 nanotubes, respectively). As expected, a monotonic behavior spanning over one order of magnitude is observed with increasing signals for increasing fractions. We then measured the fraction length distributions using AFM.

**Figure 3:**
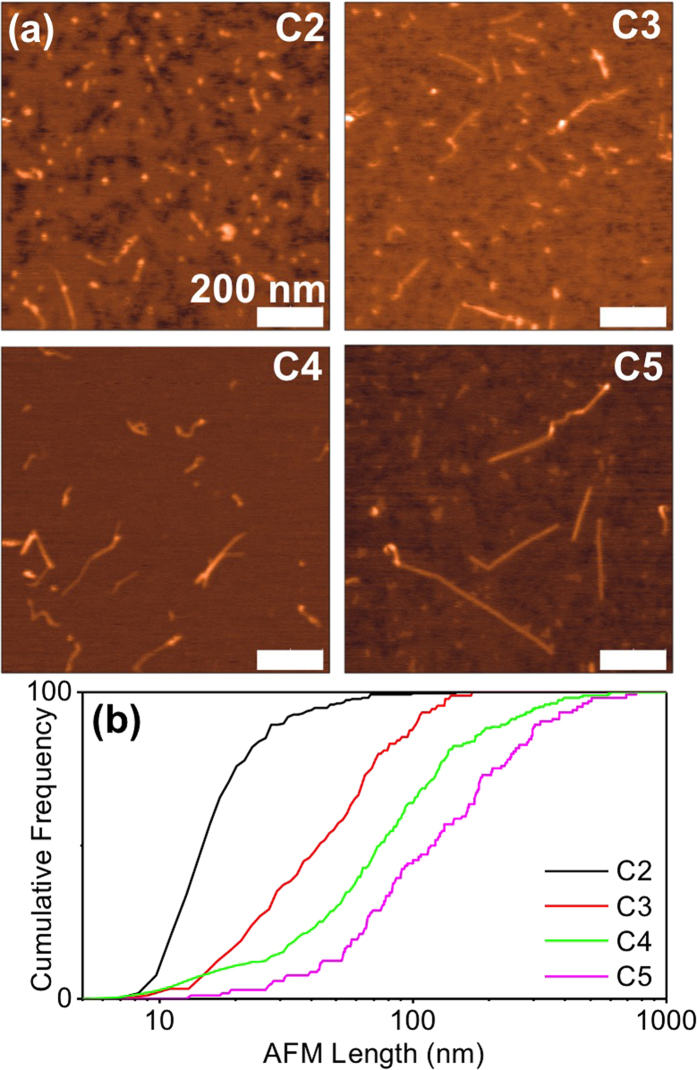
(**a**) AFM images of usCNTs in fractions C2, C3, C4, and C5; (**b**) cumulative distributions of usCNT lengths.

To this aim, a small amount of each fraction was spin-coated on a freshly cleaved mica surface. Successive washing steps using ethanol and pure-water flows were performed to remove surfactant from the surface. The samples were then naturally dried in air over night and then mounted on the AFM setup. AFM images were acquired in tapping mode in air as exemplified in Fig. 3a for fractions C2, C3, C4 & C5 (N = 218, 103, 194, 104 nanotubes, respectively). Note that no images are presented for fraction C1 where individual usCNTs could not be unambiguously identified. This is not the case of the largest fractions, such as C4 and C5, for which AFM determines the nanotube lengths Fig. 3b. For small fractions, however, such as in C2, most nanotubes appear barely elongated. In order to determine the usCNT lengths, the AFM tip size must be taken into account. In previous studies, usCNT lengths less than 10 nm were declaimed based on AFM measurements^26^,^27^. In practice, for the shortest usCNTs, AFM measurements are not only affected by the geometric size of the tip but also by the frequent tip size deterioration by e.g. surfactants leftovers which become problematic when the transverse dimensions of the studied object are in the range of the tip size^31^. To take into account this effect and determine usCNT sizes, we extracted the tip apex size for each nanotube by analyzing the apparent size of the nanotube along its transverse direction. Defining *L*_*meas*_ as the full-width-at-half-maximum (FWHM) of each usCNT profile along the elongated axis, *W*_*meas*_ as the FWHM profile perpendicular to the elongated axis (in the image plane) and *h* as the height of an imaged nanotube, we used the following expression to determine the diameter of the tip for each measurement: *D*_*tip*_ = *(W*_*meas*_ − *h)/2*. The nanotube length can thus be expressed as^26^ *L* = *L*_*meas*_ − *2D*_*tip*_. Following this protocol, nanotube lengths approaching 10 nm could be identified in fraction C2. We then built the cumulative distributions of the usCNT lengths for fractions C2, C3, C4 & C5 (Fig. 3b). Consistently with PhI imaging, longer tubes are found with increasing fraction number. Note that the distributions appear noisier than those obtained by PhI due to lower statistical data in AFM.

**Figure 4:**
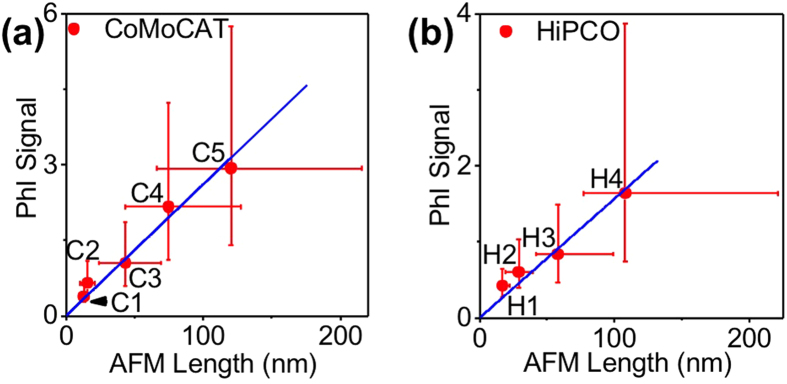
(**a**) PhI signals (excitation at 568 nm) as a function of nanotube lengths measured by AFM, determined for usCNTs in fractions C2, C3, C4, and C5; Data points represent the median values of the distributions while the bars represent 25–75 percentiles of the distributions. The blue curve is a linear fit of the data. The length corresponding to fraction C1 is an extrapolation of the measurement provided by PhI analysis using the linear fit. (**b**) Same as in (**a**) for usCNTs in H1, H2, H3, and H4 fractions.

We next compared the signal amplitudes obtained by PhI to the lengths obtained by AFM (Fig. 4a). A linear relationship is found demonstrating the quantitative character of PhI imaging for determining nanotube lengths. Interestingly, although the nanotube length measurements could not be achieved for C1 fraction by AFM, the signal amplitude obtained by PhI indicates by extrapolation that it contains nanotubes with a median length of 13 nm. This extrapolation could be performed owing to the unique sensitivity of PhI microscopy, which could reach single molecule absorption sensitivity^23^.

**Figure 5:**
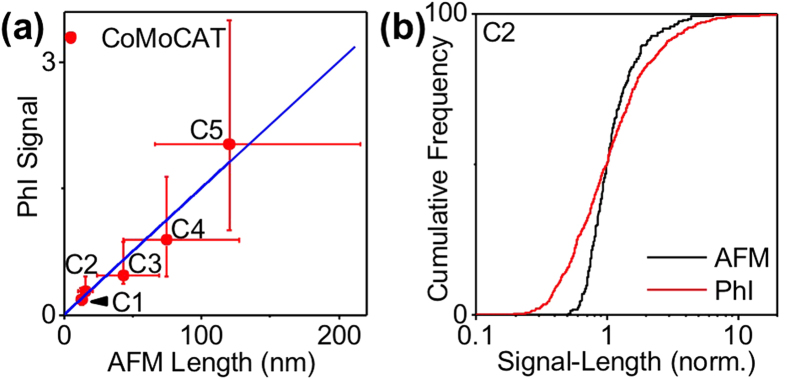
(**a**) Same as in Fig. 4(a) but for excitation at 532 nm (**b**) Comparison of PhI signals and AFM lengths distributions: both distributions where normalized by their median value for comparison.

In order to further determine whether PhI imaging can be used as a general approach for usCNT length determination irrespectively of nanotube synthesis methods or chiralities content, we performed identical experiments with HiPco samples containing a broader range of small diameter chiralities than CoMoCAT samples^32^ (Fig. 1b). Although 568 nm excitation preferentially excites (6,5) nanotubes, HiPco samples showed similar results to CoMoCAT ones (Fig. 4b, see also Fig. S2 and Fig. S3). We also used 532 nm excitation to avoid the resonant excitation of a given chirality in CoMoCAT or HiPco samples. As expected for off-resonance excitation, PhI signals are weaker than upon 568 nm excitation. A linear relationship between PhI signal amplitudes and AFM length determinations is also obtained as shown in Fig. 5a for CoMoCAT nanotubes (see also Fig. S4 and Fig. S5 for HiPco nanotubes). We further examined whether excitation at 568 nm and 532 nm would provide differences in the signal distributions by probing distinct nanotube subpopulations. To this aim, we normalized the distributions of PhI signals by their median value (Fig. S6 and S7). The normalized distributions are almost undistinguishable suggesting that the full nanotube populations are probed. In contrast, the comparison between the length distributions obtained by AFM and PhI (Fig. 5b) indicates that a narrower population of nanotubes is probed with AFM (see also Fig. S8–S9).

## Discussion

These results indicate that PhI appears as an efficient alternative to AFM measurements to determine the length content of usCNTs. AFM measurements are indeed frequently affected by adsorbed impurities, and tips can easily be degraded, in particular the smaller ones. Such measurements also need delicate sample preparations using substrates having strong interactions with the nanotubes. In addition, AFM imaging and post-acquisition analysis of usCNTs are both time demanding and tedious processes, which require experienced operators in order to precisely measure the length content of usCNT samples. In contrast, PhI measurements are fast (imaging rates of one usCNT per second has been demonstrated here) and do not require complicated sample preparation. Furthermore, PhI imaging and image analysis can easily be automated to provide length content of usCNT samples with high statistical data. In practice, PhI signals depend on several parameters e.g. laser powers, integration times and lock-in settings. PhI therefore needs calibration. This is routinely performed using standard absorbers like gold nanoparticles of known size for which the absorption cross-section is well known^18^,^33^ and can be compared to signals from usCNTs. As consequence, PhI signals will provide the absorption rate of the usCNTs. Knowing the SWCNT absorption cross-section per carbon atom^18^,^34^,^35^,^36^ one can directly access to the tube length from the PhI measurement.

**Figure 6:**
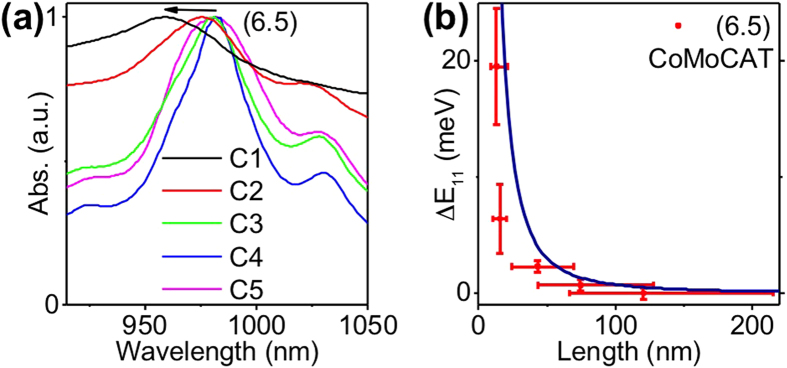
(**a**) Absorption spectra recorded around the S_11_ resonances of the (6.5) nanotubes in fractions C1 to C5; (**b**) Shifts of the S_11_ peaks of (6.5) nanotubes deduced from the absorption spectra in (**a**) as a function of median fraction lengths. The blue curve is a simple theoretical model considering the 1D box model (see text).

The estimation of the mean nanotube length in each sample could also be attempted by the analysis of the absorption spectrum of each fraction. Indeed, a blue shift of the S_11_ optical resonance was previously observed upon nanotube shortening^26^. We analyzed CoMoCAT absorption spectra recorded for different fractions (Fig. 6a). Despite a significant line broadening which complicates the determination of the peak resonance for each chirality, we observed a continuous and monotonic blue shift of the S_11_ resonance of (6.5) tubes (Δ*E*_*11*_), with the largest shift for the smallest fraction. We plotted Δ*E*_*11*_as a function of nanotube lengths obtained above (see Fig. 6b). In order to account for the confinement of excitons along the nanotube axis, the simplest model is a 1D box model leading to energy shifts of the optical transitions given by 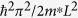 where m^*^ is the effective mass of the exciton, *L* the length of the nanotube. Using an effective mass equal to ∼0,05*m*_0_ for (6,5) nanotubes, with *m*_0_, the mass of the electron^37^, one can qualitatively reproduce the experimental data (see Fig. 6b). A quantitative agreement would require a better knowledge of the potential felt by the excitons along the nanotubes which takes into account for instance all the defect sites (including the tube ends) and annihilation processes in the tube. We note that blue shifts were not previously observed in short CoMoCAT nanotube samples^16^ which might indicate that the observed behavior is also dependent on nanotube preparations (e.g. number of defect sites, surfactants). In practice, the analysis of nanotube resonance blue shifts does not appear to provide a robust method for determining the nanotube lengths in usCNT samples.

## Conclusion

In conclusion, we achieved the preparation and separation of high quality samples of usCNTs (<150 nm) dispersed in solutions of DOC and performed the optical detection of usCNTs at the single tube level down to ~10 nm. Our detection approach is based on PhI microscopy, which probes the minute absorption of each nanotube. By comparison to AFM measurements, we show that PhI provides fast, sensitive and reliable quantification of the usCNT lengths with high statistics and minimal sample preparation. The signal amplitudes are found to be proportional to usCNT lengths. Our results demonstrate that PhI microscopy appears as an efficient detection strategy for the systematic characterization of various usCNT samples. It should be valuable for fundamental studies dedicated to the investigation of length dependent optical properties of carbon nanotubes and more generally for emerging applications of ultrashort nanotubes occurring in several fields of science.

## Methods

### Materials

CoMoCAT SWCNTs were purchased from Sigma-Aldrich and HiPco SWCNTs (197.5) from Rice University. All nanotubes samples were used without further purification for preparing usCNTs. All other chemicals were purchased from Sigma-Aldrich otherwise stated.

### usCNT preparation

CoMoCAT or HiPco solutions were prepared by dispersing 30 mg of raw nanotubes in 10 mL of milli-Q water containing 0.3 wt% sodium deoxycholate (DOC) using a tip sonicator in pulsed operation for 5 h (output at 45 W, pulse 0.5 s and pause 0.2 s). The vials containing the solutions were kept in an ice bath during sonication to avoid overheating that could degrade DOC. After sonication, the samples were gently centrifuged for 30 min (3500 rpm, Eppendorf Centrifuge 5804 R) to remove large bundles and insoluble materials. Supernatants (typically 9 mL) were then collected and diluted up to 20 mL with a 0.3 wt% DOC solution. They were then ultra-centrifuged for 2 h (45000 rpm, Beckman Coulter) to remove remaining bundles and catalyst particles. This process was repeated twice, and the final supernatants were collected for length separation experiments.

### Density gradient ultracentrifugation (DGU)

Density gradient solutions were prepared via subsequently stacking four layers of 5 mL of 60 wt%, 10 wt%, 7.5 wt%, and 5 wt% iodixanol solution (OptiPrep, Sigma-Aldrich) from bottom to top in 26.3 mL polycarbonate centrifugal tubes (Beckman Coulter). In order to achieve length separation, a volume of 5 mL sonication-cut SWCNT suspension was placed on top of the density-gradient iodixanol solution and centrifuged at a speed of 45000 rpm for 3 h at 4 °C. After ultracentrifugation, SWCNTs with different lengths are found distributed along the density gradient of the solvent because of different sedimentation velocities. Longer SWCNTs are located nearer to the bottom of the centrifugal tube, and shorter SWCNTs are nearer to the top. The solution containing SWCNTs in the centrifugal tube was then carefully divided and collected as fractioned samples (0.9–1 mL each fraction) from top to bottom. For long-term storage, the concentration of DOC in all fractions was raised up to 1 wt% to maintain individual dispersion of nanotubes and samples are stored at 4 °C.

### Absorption spectra

Absorption spectra were recorded with a Varian Cary 5000 UV-Vis-NIR absorption spectrometer from 400 to 1400 nm at ambient temperature in 1 cm cuvettes. A reference spectrum measured using a solution of pure 1 wt% DOC was subtracted from all nanotube spectra.

### AFM imaging

A drop (20 μL) of usCNT solution was spin-coated on a freshly cleaved mica surface using gradual spinning speeds (500–2000 rpm/1 min, 2000 rpm/1 min, 2000–5000 rpm/1 min, 5000 rpm/4 min). The sample was then washed with ethanol and pure-water several times in order to remove surfactants from the surface and dried in air over night. For imaging, a Veeco BioScope AFM was used in tapping mode in air (typical image scale 1 × 1 μm). Cantilever tips with a theoretical radius of 7 nm were generally employed. Additionally, a FastScan AFM with 5 nm theoretical radius cantilevers could also be employed in force-tapping mode.

### Sample preparation for PhI imaging

A drop (20 μL) of usCNTs suspension was spin-coated on a glass cover slip pre-coated with a thin polyvinyl alcohol layer (3 wt%). The sample was then sandwiched by another cover slip after having added 5 μL of viscous silicone oil. The sample was then mounted on the PhI setup to be imaged.

## Additional Information

**How to cite this article**: Gao, Z. *et al.* Optical detection of individual ultra-short carbon nanotubes enables their length characterization down to 10 nm. *Sci. Rep.* **5**, 17093; doi: 10.1038/srep17093 (2015).

## Supplementary Material

Supplementary Information
