# Supplementary material for: Optical detection of individual ultra-short carbon nanotubes enables their length characterization down to 10 nm
Source: Sci Rep. 2015 Nov 25;5:17093. doi: 10.1038/srep17093 (PMC4658524; doi:10.1038/srep17093)
Supplement: Supplementary Information [file srep17093-s1.pdf]

Supplementary Information

Optical detection of individual ultra-short carbon nanotubes enables their length characterization down to 10 nm

Zhenghong Gao[a,b,#], Laura Oudjedi[a,b,#], Romain Faes[c], Fabien Moroté[d], Christèle Jaillet[c], Philippe Poulin[c], Brahim Lounis[a,b] and Laurent Cognet[a,b]*

[#] These authors contribute equally

* Corresponding should be addressed to: Laurent.cognet@u-bordeaux.fr

[a] Univ. Bordeaux, LP2N, F-33405 Talence, France;

[b] Institut d'Optique & CNRS, LP2N, F-33405 Talence, France;

[c] CRPP, CNRS, Pessac, France;

[d] LOMA, CNRS & Univ. Bordeaux, F-33405 Talence, France

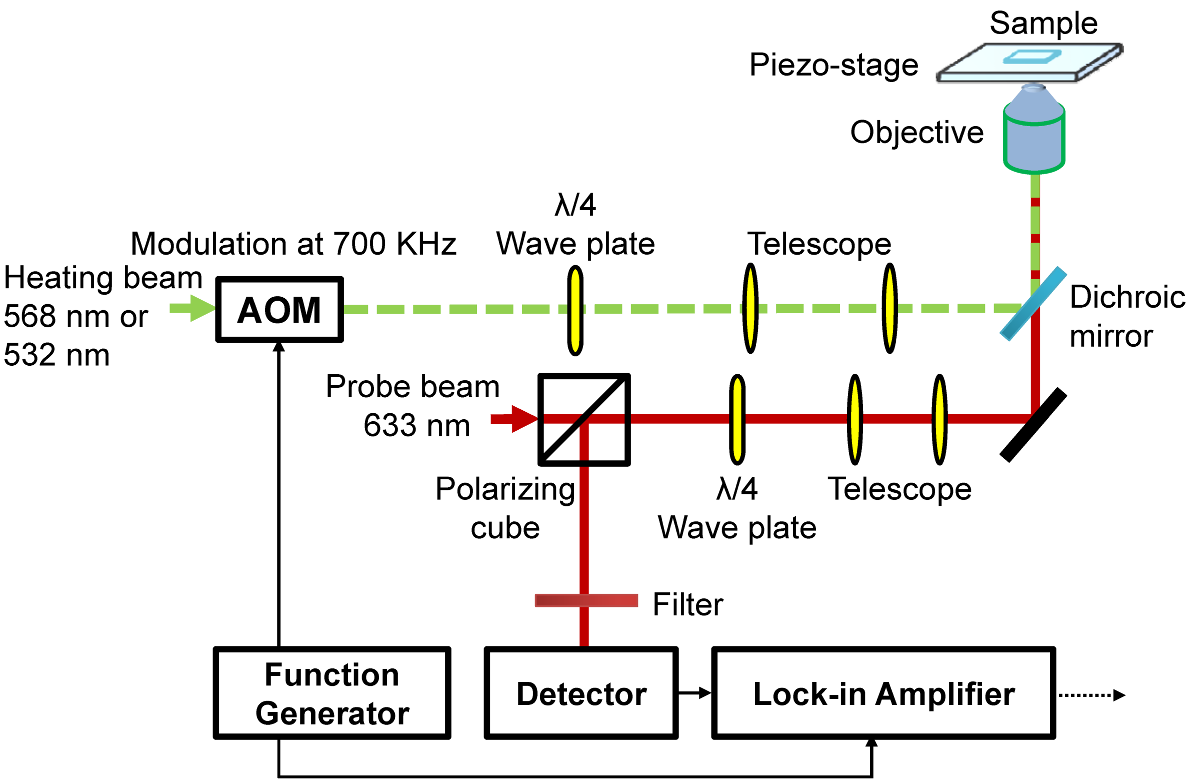

Figure S1. Optical setup of PhI.

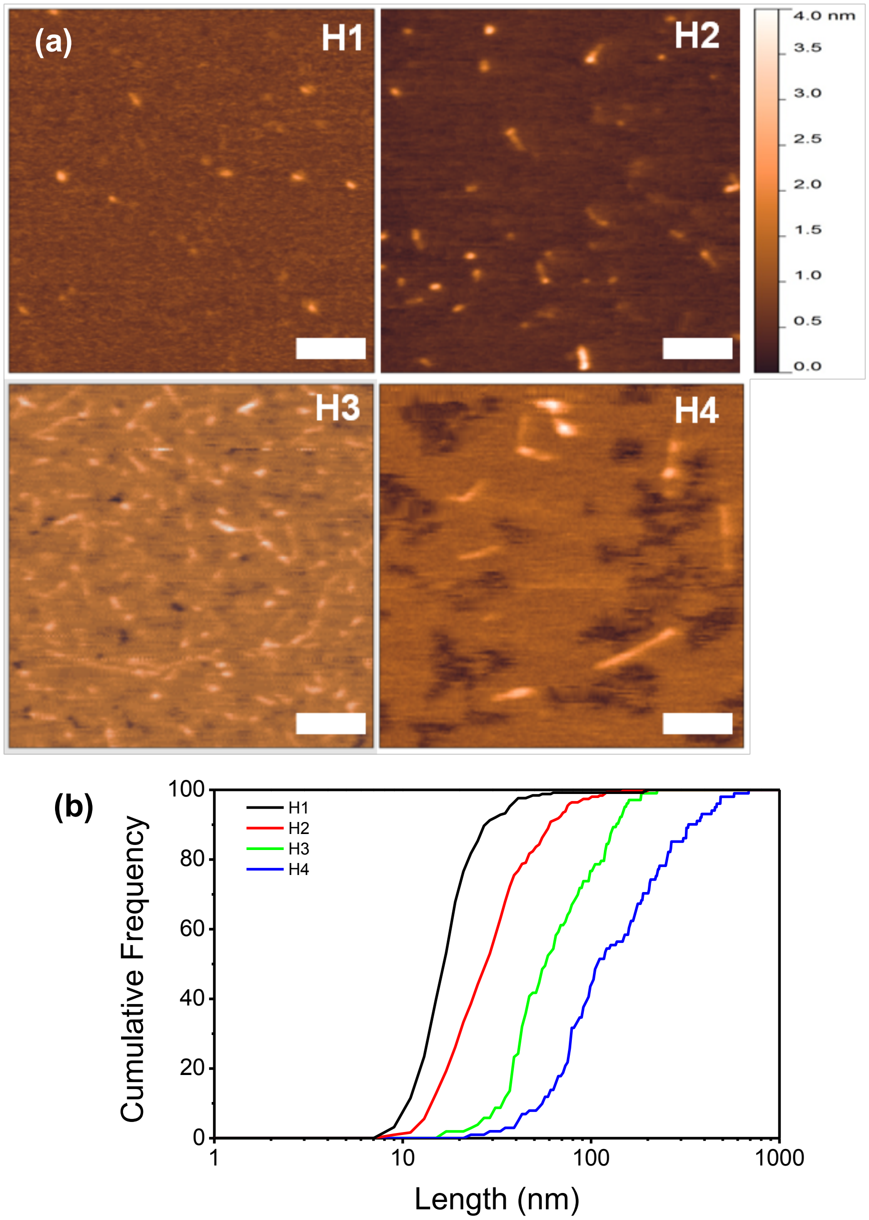

Figure S2. (a) AFM images of usCNTs in H1, H2, H3, and H4 samples and (b) cumulative distributions of the length measured based on AFM images. N =252, 306, 103, 101 respectively. Scale bars are 200 nm.

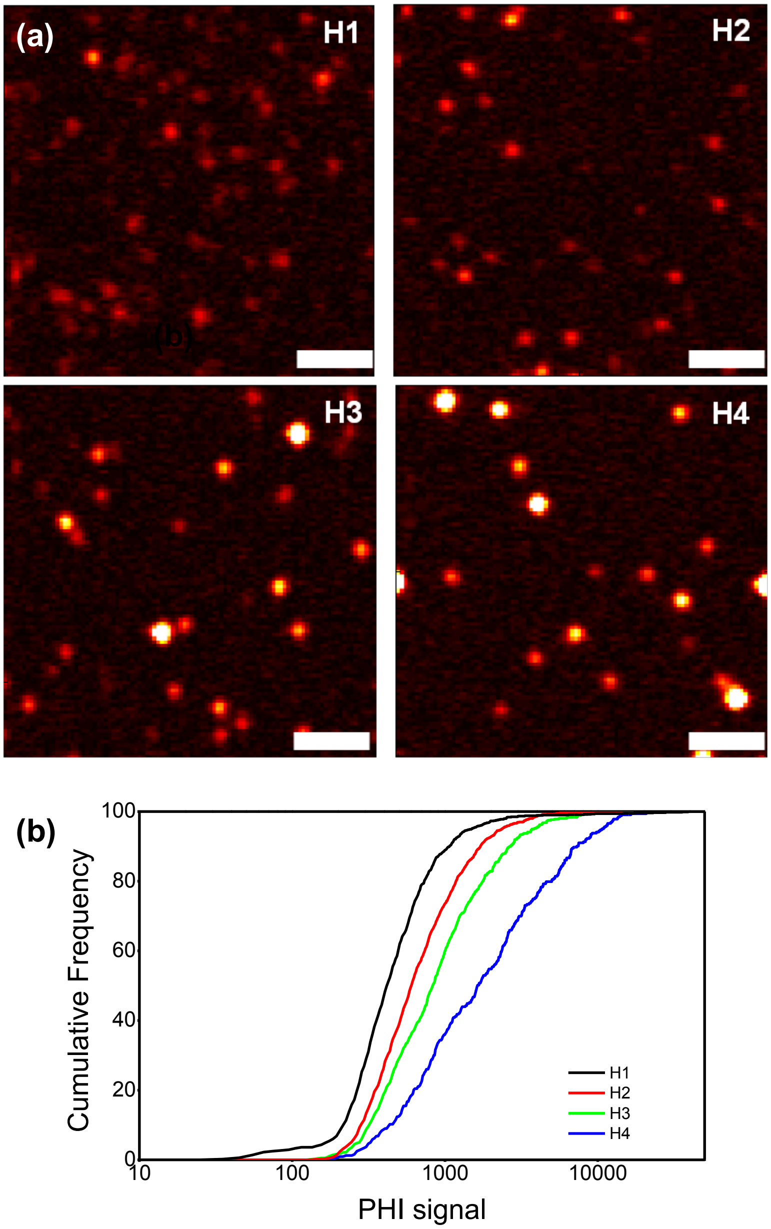

Figure S3. (a) PhI images of usCNTs in H1, H2, H3, and H4 samples and (b) cumulative distributions of the signals measured based on PhI images (excitation at 568 nm). N = 988, 1300, 560, 323 respectively. Scale bars are 2 μm. Images have same intensity scales.

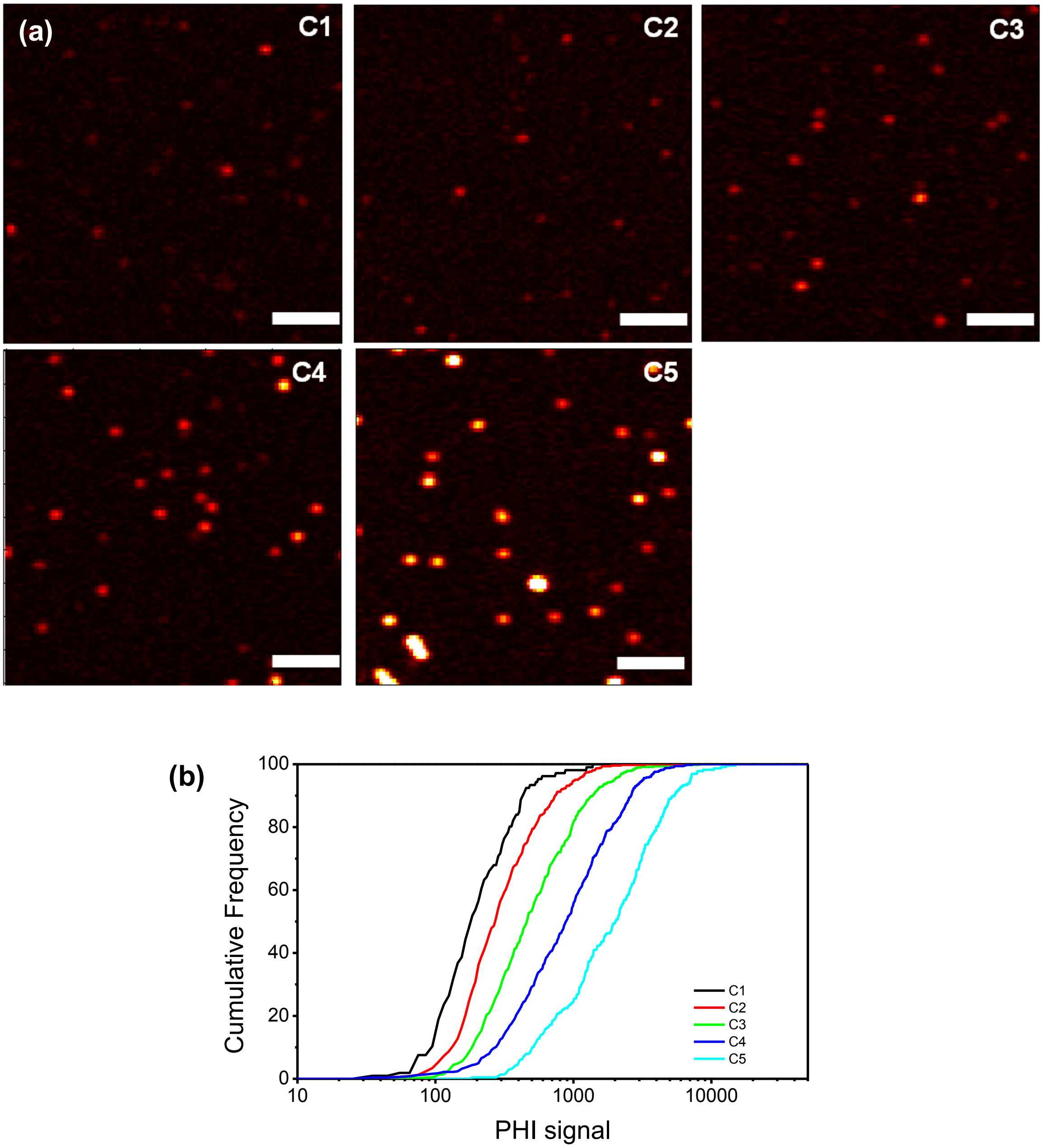

Figure S4. (a) PhI images of usCNTs in C1, C2, C3, C4, and C5 samples and (b) cumulative distributions of the signals measured based on PhI images (excitation at 532 nm). N = 988, 469, 472, 503, 227 respectively. Scale bars are 2 μm. Images have same intensity scales.

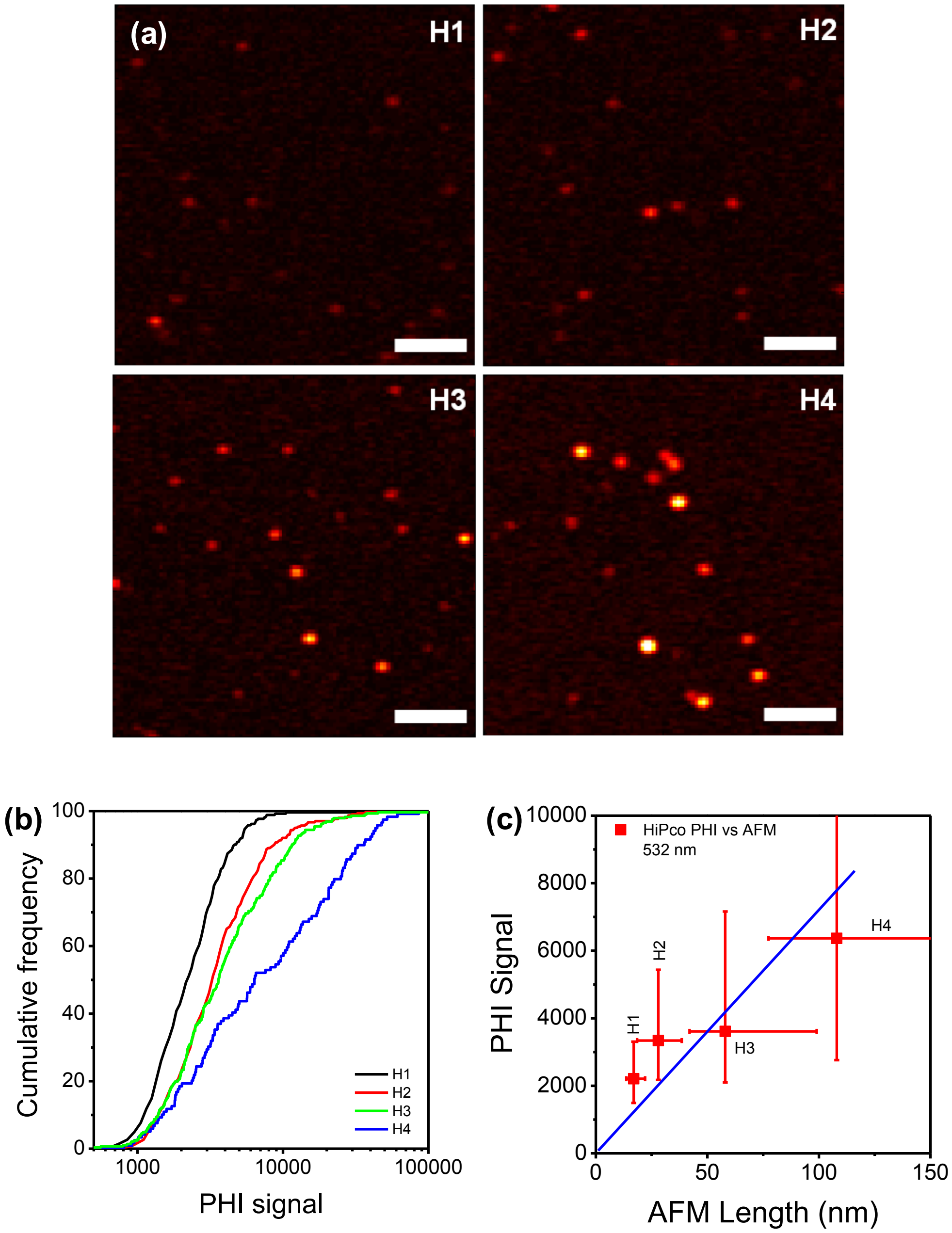

Figure S5. (a) PhI images of usCNTs in H1, H2, H3, and H4 samples and (b) distributions of the signals measured based on PhI images for H1, H2, H3, and H4 samples (excitation at 532 nm). N = 262, 301, 287, 119 respectively. Scale bars are 2 μm. Images have same intensity scales. (c) PhI signals (excitation at 352 nm) as a function of AFM lengths, determined for usCNTs. Data points represent the median values of the distributions while the bars represent 25-75 percentiles of the distributions. The blue curve is a linear fit of the data.

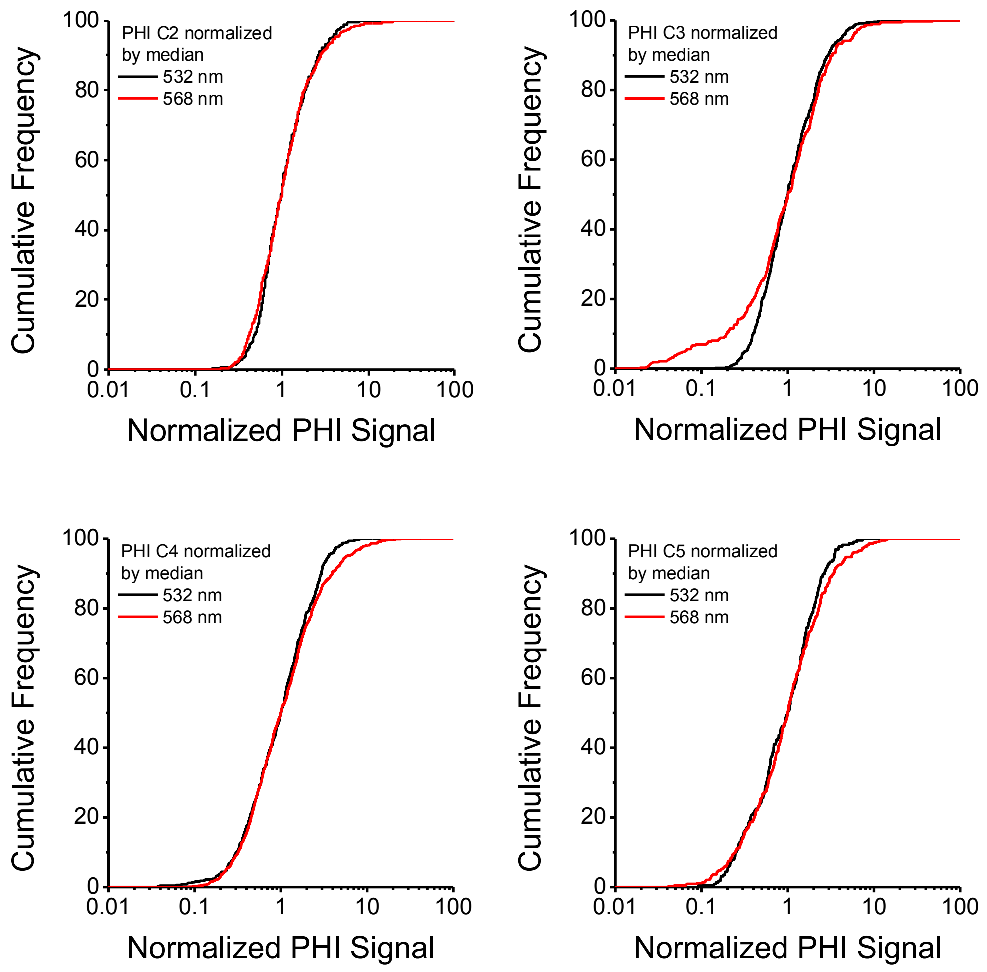

Figure S6. Normalized PhI signal cumulative distributions for CoMoCAT samples (C2, C3, C4, C5 fractions) for comparison between excitation at 568 nm and 532 nm.

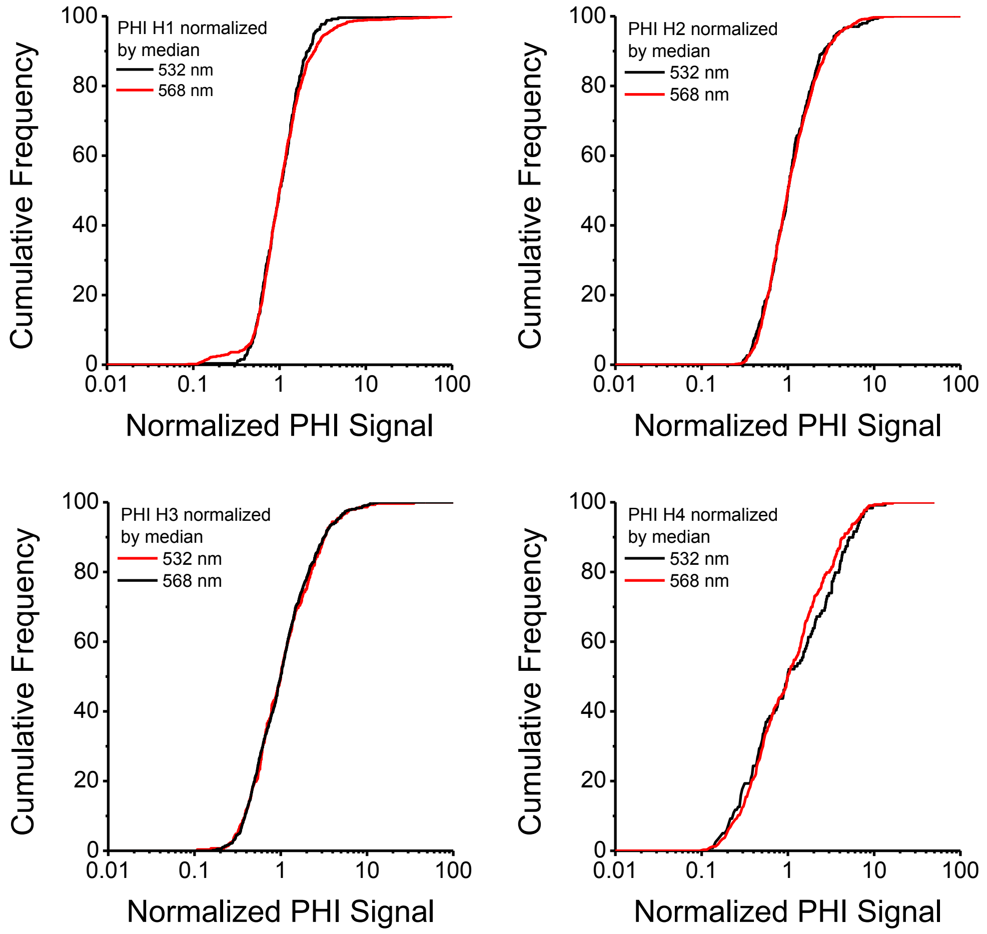

Figure S7. Normalized PhI signal cumulative distributions for HiPCO sample (H1, H2, H3, H4) for comparison between excitation at 568 nm and 532 nm.

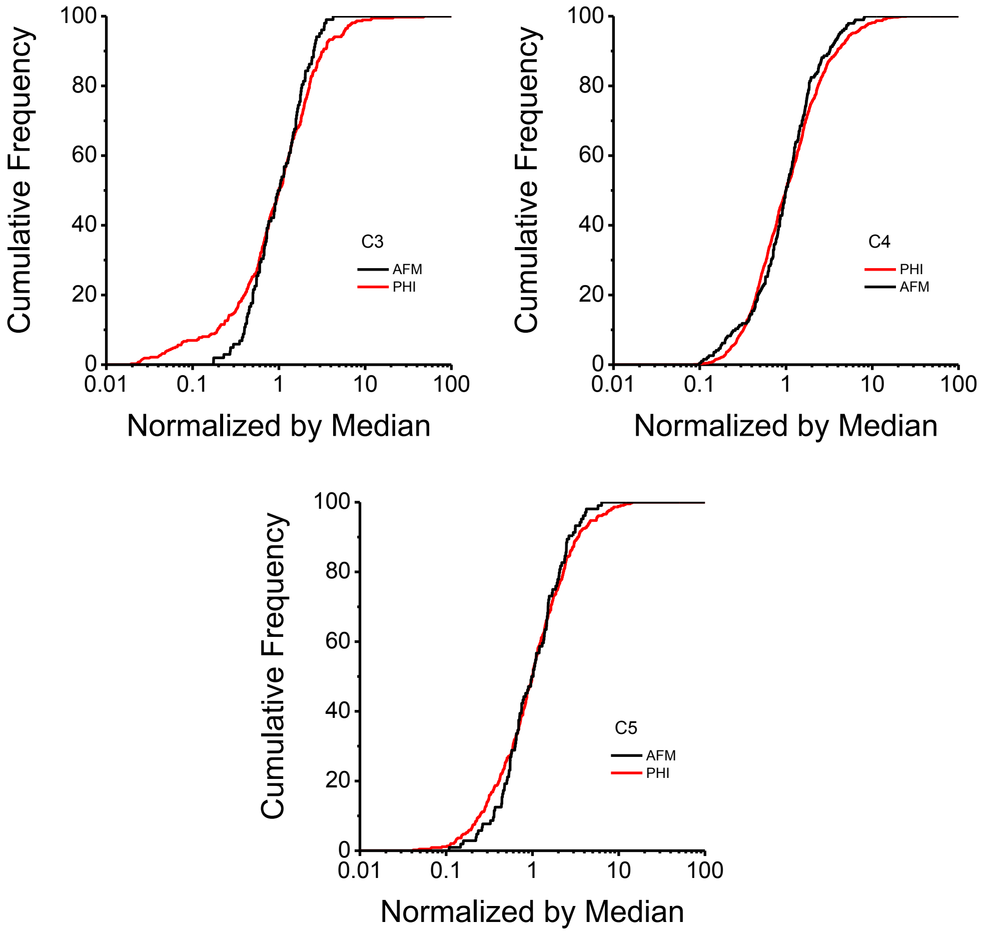

Figure S8. PhI signal and AFM length cumulative distributions for CoMoCAT sample (C3, C4, C5) are normalized for comparison.

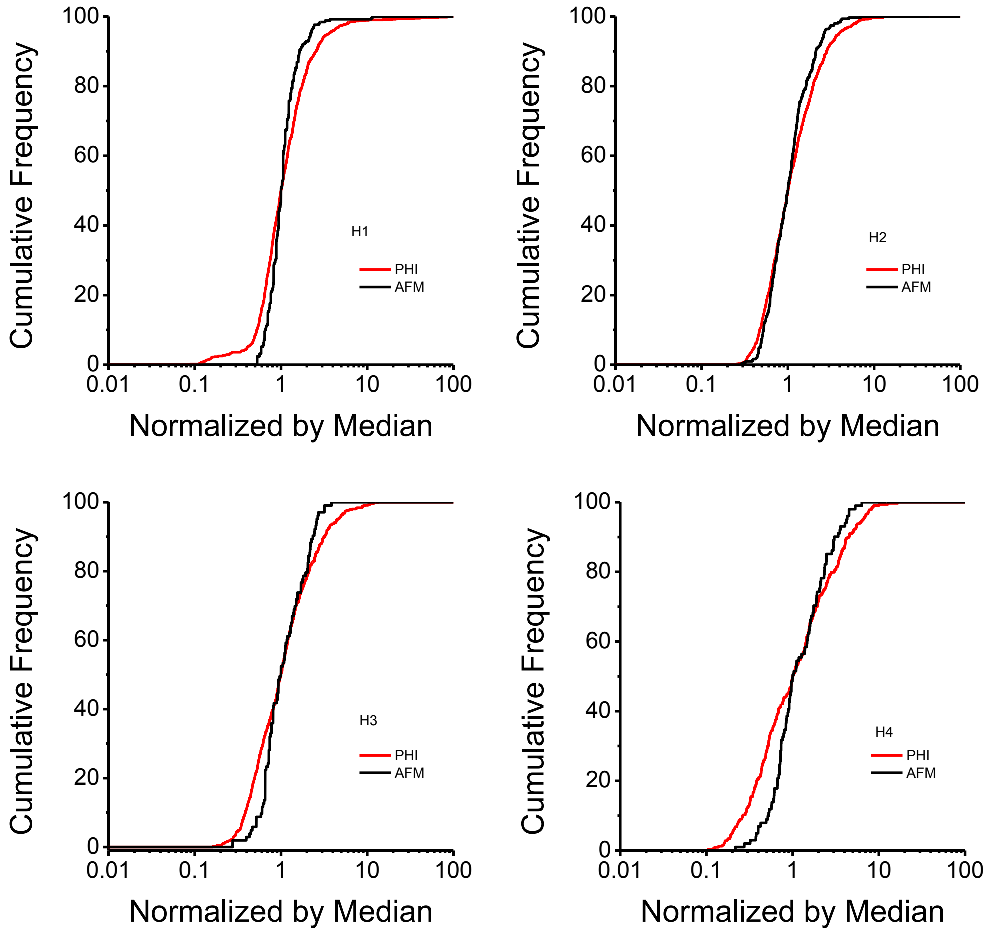

Figure S9. PhI signal and AFM length cumulative distributions for HiPCO sample (H1, H2, H3, H4) are normalized for comparison.
